# Privacy protections to encourage use of health-relevant digital data in a learning health system

**DOI:** 10.1038/s41746-020-00362-8

**Published:** 2021-01-04

**Authors:** Deven McGraw, Kenneth D. Mandl

**Affiliations:** 1 Ciitizen, Palo Alto, CA, USA; 2 Boston Children’s Hospital, Harvard Medical School, Boston, MA USA

**Keywords:** Social sciences, Policy

## Abstract

The National Academy of Medicine has long advocated for a “learning healthcare system” that produces constantly updated reference data during the care process. Moving toward a rapid learning system to solve intractable problems in health demands a balance between protecting patients and making data available to improve health and health care. Public concerns in the U.S. about privacy and the potential for unethical or harmful uses of this data, if not proactively addressed, could upset this balance. New federal laws prioritize sharing health data, including with patient digital tools. U.S. health privacy laws do not cover data collected by many consumer digital technologies and have not been updated to address concerns about the entry of large technology companies into health care. Further, there is increasing recognition that many classes of data not traditionally considered to be healthcare-related, for example consumer credit histories, are indeed predictive of health status and outcomes. We propose a multi-pronged approach to protecting health-relevant data while promoting and supporting beneficial uses and disclosures to improve health and health care for individuals and populations. Such protections should apply to entities collecting health-relevant data regardless of whether they are covered by federal health privacy laws. We focus largely on privacy but also address protections against harms as a critical component of a comprehensive approach to governing health-relevant data. U.S. policymakers and regulators should consider these recommendations in crafting privacy bills and rules. However, our recommendations also can inform best practices even in the absence of new federal requirements.

## Introduction

The National Academy of Medicine has long advocated for a “learning healthcare system” that produces constantly updated reference data during the care process^1^. Moving toward a rapid learning system to solve intractable problems in health demands a balance between protecting patients and making data available to improve health and health care. Since much of what impacts an individual’s health and wellbeing occurs outside of a doctor’s office or hospital^2^, a rapid learning health system also requires data generated outside of traditional healthcare.

This paper comprehensively explores the growing U.S. health data landscape and the privacy risks and innovation obstacles raised by under-regulation of this data. We propose a multi-pronged approach to protecting “health relevant” data while promoting and supporting beneficial uses and disclosures essential to improving health and health care. A roadmap of the key points explored in this paper can be found at Box 1.

Box 1 Overview

- A wide array of information about individuals is health-relevant.
- Health information can have beneficial and detrimental impacts for individuals and populations, depending on use.
- HIPAA provides limited coverage of health data, including data shared by consumers with third party applications.
- The Federal Trade Commission regulates companies’ use of health data but not through comprehensive rules.
- Other U.S. and international laws provide only some protection for health data in the U.S.
- Congress is considering privacy legislation, but the bills have significant shortcomings for protecting health data. They:

–Overrely on providing individuals with greater notice and consent, which feels empowering for consumers but shifts to them the burden for protecting data.
–Overvalue de-identification or pseudonymization as privacy measures, providing zero protections for de-identified data notwithstanding potential re-identification risk and concerns about commercialization.
–Do nothing to encourage responsible uses of health data to improve individual and population health, notwithstanding significant shortcomings in U.S. health and health care.
–Focus largely on entities not covered by HIPAA, though HIPAA urgently needs reevaluation given current practices.

- COVID-19 responses sharply illustrate the tension between beneficent data use and privacy incursions.
- The dual requirements to both protect individuals and assure data availability call for comprehensive policies governing all entities collecting and using health information, whether covered by HIPAA or not. Legislation and company best practices should draw from HIPAA’s framework and FTC consumer privacy recommendations and include:

–Increased transparency and choice for consumers.
–Limitations on how health data can be collected, used, and disclosed versus relying only on consent.
–Mechanisms to assure beneficial uses of health relevant data, e.g., independent data ethics boards, health trusts, impact assessments, and accountable data custodians.
–Strengthened remedies for harms incurred from malevolent uses of health data.
–Accountability for uses of de-identified data.

### Defining health data

Though traditionally, the term “health data” has referred to information produced and stored by healthcare provider organizations, vast amounts of *health-relevant data* are collected from individuals and entities elsewhere, both passively and actively. Much of data beyond Category 1 in Box 2 is outside of the scope of comprehensive health privacy laws in the U.S.

Recently, concerns about whether existing privacy laws provide sufficient protections for health-relevant data have motivated Congress and state legislatures to propose legislation to fill the gaps^3^. These concerns have been exacerbated by revelations about how the largest information technology companies collect, use, and share personal data^4,5^ and these companies’ increasing activities in health care^6^.

The tension between protecting privacy while promoting more widespread access to health-relevant data is not new. Data produced by the healthcare system (Category 1) has been difficult to access and marshal for health reform, to protect public health, to underpin discoveries, or to expand the evidence base for health and wellness interventions^7^. Yet recent new federal initiatives aimed at increasing access to Category 1 data—particularly with respect to sharing this data with consumer-facing applications—were met with fierce resistance as privacy concerns were raised^8^.

At the same time, nontraditional health-relevant data (Categories 2–4), often equally revealing of health status, are in widespread commercial use and, in the hands of commercial companies, largely unregulated—yet often less accessible by providers, patients and public health for improving individual and population health^9^. The following examples illustrate this tension. There is increasing recognition that social determinants of health (sometimes discernable from data in Categories 3–4) can be highly influential in health and wellness and the costs of both^2,10^. On the one hand, these data are sensitive because of stigma, health, and financial implications associated with having limited resources. On the other hand, they could be used to improve the health of individuals by identifying those most in need of supports and services. However, in the absence of controls companies could use these data perversely, for example, to avoid locating in neighborhoods perceived to be more costly, or to avoid insuring populations with the highest risks from social determinants. Other types of data in Categories 3 and 4 may not on the surface appear to be health-relevant but could be used to make powerful inferences about an individual’s or a population’s health, e.g. homeownership and job status are predictive of medication adherence^11^. Such information either could be used to target resources toward improving adherence or to penalize individuals unlikely to take their medications.

To date, U.S. laws governing health data and new legislative proposals tend to focus more on privacy by limiting or controlling access to health-relevant data than on assuring its availability for uses that could improve individual and population health. Lacking are multifaceted policy solutions incorporating protections for health-relevant data while stimulating and encouraging responsible uses for transforming healthcare into a more data-driven enterprise. Necessary protections for health-relevant data also must go beyond a pure privacy focus and extend to preventing or penalizing uses that could harm individuals and populations. Here, we address both privacy protections but also potential data-related harms as a critical component of a comprehensive approach to governing health-relevant data.

Box 2 Major categories of health-relevant data with examples

1. **Category 1. Health Care System Generated**. Electronic medical record data, prescriptions, laboratory data—including molecular “omics” data, pathology images, radiography, payor claims data.
2. **Category 2. Consumer Health and Wellness Industry Generated.** Wearable fitness tracking devices, medical wearables such as insulin pumps and pacemakers, medical or health monitoring apps, patient-reported outcome surveys, direct-to-consumer tests (including DNA analysis) and treatments.
3. **Category 3. Digital Exhaust Generated as a Byproduct of Consumers’ Daily Activities.** Social media posts, Internet search histories, location and proximity data.
4. **Category 4. Non Health Demographic, Social, and Economic Sources.** Race, gender, income, credit history, employment status, education, level, residential zip code, housing status, census records, bankruptcy and other financial records, grocery store purchases, fitness club memberships, voter registration.

### U.S. Federal privacy protections for health-relevant personal data

#### The limitations of HIPAA

The privacy, security, and breach notification regulations promulgated under the Health Insurance Portability and Accountability Act of 1996 (HIPAA)^12^ and the Health Information Technology for Economic and Clinical Health Act of 2009 (HITECH)^13^ provide a comprehensive set of protections—but only for data within the health care system. Like many U.S. laws, HIPAA is a sectoral law, covering only certain types of entities. For the most part, HIPAA does not extend to organizations and businesses outside of the traditional health care ecosystem. (See Supplementary Discussion for a brief overview of HIPAA and other laws governing data in the health care system.) Though many may think of HIPAA as only applying to Category 1 data, once health-relevant data—in any of the four categories in Box 2—are collected within an entity covered by HIPAA, those data will be covered by HIPAA’s protections. Specifically, all information that is identifiable “protected health information” is covered^14^, and this includes information that may not look like health data (such as in Category 4) but is used in a way that makes it “related to health and health care.” However, since HIPAA’s coverage is about “who” holds the data, but not what type of data, much of the health-relevant data collected today is collected by entities outside of HIPAA’s coverage bubble and thus resides outside of HIPAA’s protections^15–17^.

An increasingly important example of information leaving HIPAA’s coverage is when a consumer uses a third party health application (app) to obtain Category 1 data for personal use. Health apps used by consumers are frequently hosted by third parties and may share data further, with little transparency to users. Most are not covered by HIPAA. An analysis of 10 apps (two of them intended to enable women to track menstrual cycles and predict ovulation times) found they transmitted data on user activities in the app to 70 different third parties involved in advertising and profiling, without explicit consent from the users^18^. Another study examining 14 health and nutrition apps, including apps tracking medication use, migraines, and sleep, and some helping to manage diabetes, found that all but one (the Apple Health App) shared data with third parties without full transparency to the user^19^. A cross-sectional study of 36 apps for depression and smoking-cessation researchers found that 29 transmitted data to services provided by Facebook or Google, but only 12 accurately disclosed this in a privacy policy^20^.

Privacy concerns over consumer apps nearly halted new federal initiatives that require health care providers and health plans to share more health information with patients. As directed by the 21st Century Cures Act^21^, the federal Office of the National Coordinator for Health IT (ONC) recently finalized rules prohibiting “information blocking,” which includes the failure to share health information with individuals or health apps chosen by those individuals^22^. ONC’s rules also require certified electronic health records (EHRs) used by health care providers to offer open, standard application programming interfaces (APIs), specifically the SMART on FHIR API^23^ and the SMART/HL7 Bulk Data API^24^^,25^, to facilitate seamless digital data sharing of electronic health record data, including with individuals and their chosen health apps^24^^,25^. Demonstrating a similar commitment to greater data sharing, particularly with individuals, the Centers for Medicare and Medicaid Services (CMS) now requires health plans under its purview to share claims data with subscribers and hospitals to send alerts to physicians when their patients have been hospitalized^26^.

When CMS and ONC initially proposed these rules and sought public comment, they were sharply criticized by health care provider organizations and by a major vendor of provider EHR systems for promoting the sharing of sensitive clinical and claims data—data in Category 1 - with consumer-facing apps without adequately addressing privacy concerns^27^. Although neither CMS nor ONC has authority to regulate consumer-facing tools, the critics capitalized on a significant gap in U.S. health privacy protections^28^.

#### FTC jurisdiction and other protections

The new federal rules on interoperability and information blocking facilitate patient-mediated data flows, sending EHR data (Category 1) across an API, leaving a HIPAA-covered entity and entering a consumer-controlled app. As the data traverse the API, the regulatory authority instantaneously shifts from the HHS Office for Civil Rights (OCR), which enforces HIPAA, to the Federal Trade Commission (FTC)^29^. The FTC has the most enforcement power over privacy in the U.S. through Section 5(a) of the FTC Act (FTCA), which broadly prohibits “unfair or deceptive acts or practices in or affecting commerce”^30^. The FTCA applies to most entities engaged in commerce, including developers and marketers of mobile health technologies, social media sites, and technology companies. Generally, the FTC’s Section 5 authority does not extend to nonprofit entities or insurance companies (https://www.ftc.gov/news-events/media-resources/what-ftc-does), and there are exceptions related to banks, savings and loans, federal credit unions, and common carriers such as airlines^31^.

In the context of privacy, the FTC has translated its unfair and deceptive trade practices authority by, for example, requiring companies covered by the FTCA to honor their commitments set forth in privacy policies and service and to adopt reasonable security safeguards^32^. Further, the Commission has brought numerous cases against businesses covered by the FTCA for failing to protect consumers from companies’ deceptive and unfair practices with regard to their health data and failing to have reasonable and appropriate data security practices regarding that data^33^.

Entities not covered by the FTCA (for example, nonprofit entities and insurance companies) may be regulated regarding privacy and security only if covered by another federal law (HIPAA, for example) or by state law. Ironically, this means that in terms of federal privacy protections, an app offered by a nonprofit company outside of the health care system (for example, offered by a patient advocacy organization) might offer the least accountability to consumers. Of note, the FTC also administers breach notification requirements enacted by Congress in the Health Information Technology for Economic and Clinical Health Act (HITECH) (see Supplementary Discussion) and applicable to “personal health records,” which are health records maintained by or primarily for individuals^34^, and “related apps”^13^.

The FTCA is broadly applicable to most companies collecting health-relevant data, and the FTC has taken enforcement action against developers of mobile health apps^35^. However, there is a perception that these protections—because they are not established in comprehensive regulations similar to the HIPAA rules—are not sufficient to protect health data^36^. Notwithstanding the breadth of FTC’s authority, its recent settlement with Facebook regarding a number of alleged violations of the FTCA has generated doubts about whether the FTC is equipped to take on takes this enforcement role outside of HIPAA’s boundaries^37,38^. Concerns have also been raised that the FTC currently lacks sufficient resources to enforce privacy protections for health-relevant data at scale.

Other federal statutes extend some privacy protections for personal data, which could include health-relevant data, in particular contexts^39^ (See Supplementary Table 1 for a brief summary of some federal laws that extend protections for personal data). State privacy laws protecting health and personal data often are more protective than federal law^40,41^. For example, HIPAA does not preempt state laws that are more protective of privacy^42^. To help resolve privacy concerns, a number of organizations have proposed voluntary privacy frameworks for health data. Voluntary commitments made by companies subject to the FTCA can be enforced by the FTC (See Box 3 for a summary of these efforts).

Finally, international laws also can affect protections for U.S residents if global companies governed by international laws decide to apply those heightened protections to all of their customers. For example, the Global Data Protection Regulation (GDPR), which went into effect in May of 2018, covers all data “controllers” and “processors” in the European Union (EU). It also includes entities not located in the EU but who offer goods and services to EU residents or monitor the behavior of EU data subjects within the EU^43^. Commitments U.S. companies make to their U.S. customers to comply with the GDPR can be enforced by the FTC.

Box 3 Summary of health data best practice frameworks Detailed, voluntary privacy and security best practice frameworks have been proposed, including by the Center for Democracy and Technology (CDT) and the eHealth Initiative (eHI) (https://www.ehidc.org/resources/draft-consumer-privacy-framework-health-data), the CARIN Alliance (https://www.carinalliance.com/our-work/trust-framework-and-code-of-conduct), the Consumer Technology Association (https://www.cta.tech/cta/media/policyImages/policyPDFs/Guiding-Principles-on-the-Privacy-and-Security-of-Personal-Wellness.pdf), the American Medical Association’s Xcertia Initiative (https://xcertia.org/the-guidelines/), and Patient Privacy Rights (https://papers.ssrn.com/sol3/papers.cfm?abstract_id=3439701). These best practices, if publicly attested to by companies subject to the FTCA, can be enforced by the FTC (such as if a company publicly commits to good data practice but doesn’t actually follow that practice). In addition, in 2016 the ONC published a Model Privacy Notice intended to help consumers compare privacy policies across consumer health applications (https://www.healthit.gov/sites/default/files/2016_model_privacy_notice.pdf (02 Dec 2016)).

These best practice frameworks and the model notice differ in their intended uses and level of detail, but there are similarities. Each address issues of transparency to consumers and when consent for data collection, use, or disclosure is necessary. Each provide consumers with rights such as the right to access and request amendments to data. Most cover only identifiable data, although at least one, the CARIN Trust Framework, requires transparency for uses of less identifiable or “de-identified” data. One, Xcertia, focuses also on the reliability of the health data or advice dispensed by consumer-facing services. The framework from the Consumer Technology Association recommends that personal wellness data not be knowingly used or disclosed “in ways that are likely to be unjust or prejudicial” to consumers and encourages companies to periodically review algorithms or automated decision methodologies to guard against the creation of unjust or prejudicial outcomes for subgroups. The CDT/EHI framework would place collection, use, and disclosure limitations on health data and require that automated, algorithmic or artificial intelligence systems be designed and implemented in ways to mitigate bias.

### Reevaluating HIPAA

The presumption has been that, at least with respect to Category 1 data, the U.S. has sufficient protections in HIPAA, but that presumption appears to be fading. Some have questioned whether HIPAA is still protective in an increasingly digital era^44^. The more the public learns about what HIPAA allows, the less satisfied they are with the “protections” afforded by the law. For example, entities covered by HIPAA frequently sell data that are de-identified per HIPAA standards but still can be linked to create health profiles of individuals^45^.

In particular, HIPAA’s sufficiency is being questioned when it comes to sharing health-relevant data with large technology companies. After years of periodic experimentation, major technology companies are retooling business models to address need in health care (https://www.cbinsights.com/research/apple-healthcare-strategy-apps/). In many cases, these initiatives involve contracting with health care system actors to improve how these actors deliver health care^46–49^. Technology companies have the potential to bring needed resources and innovation into health and health care^50^. However, the tech companies’ history of ubiquitous data collection and tracking of consumers has generated public backlash^51^. Facebook^52^, Google^53^, and Twitter (https://help.twitter.com/en/information-and-ads#10-08-2019) have had substantial lapses in protecting personal information, generating public doubt that these companies can yet be trusted to responsibly handle health-relevant data^54^. A whistleblower’s alarm^55^ over an arrangement between Google and Ascension Health to facilitate data analytics for Ascension caused an uproar, triggering investigations by the U.S. Department of Health and Human Services (HHS) to assure the arrangement complied with HIPAA regulations^56^. In the arrangement, Google is a vendor to Ascension and covered under HIPAA as a business associate. Consequently, Google must abide by the same rules that govern health care providers and health plans, plus any additional Ascension may have imposed as part of the business associate agreement. Nevertheless, this arrangement sparked a public conversation, suggesting public dissatisfaction with such data arrangements even when they are in compliance with HIPAA.

### COVID-19: the perfect storm

COVID-19 may perfectly illustrate the conundrum between protecting health information and ensuring its availability to meet the challenges posed by a significant global pandemic. U.S. lawmakers have used enforcement discretion to relax existing health privacy laws to stimulate more widespread reporting of relevant COVID-19-related data to federal and state public health authorities^57^. Public health experts have published best practices to enable existing health information exchange networks—built to facilitate digital data sharing among health care providers for treatment purposes—to be rapidly leveraged for public health reporting^58^.

But efforts from major technology companies to assist in fighting the pandemic have been met with skepticism. Verily’s establishment of community testing sites—and an online site to screen people for eligibility for a test—was initially met with criticism from privacy advocates^59^. Public health experts (https://apps.npr.org/documents/document.html?id=6877567-Bipartisan-Public-Health-Leaders-Letter-on) are calling for robust contact tracing to combat COVID-19 and help states and localities begin to safely re-open public spaces^60^. China and South Korea have mandated public use of contact tracing technologies, with few privacy controls^61^; other countries are also adopting contact tracing technologies^62^. However, in the U.S., states and localities have been slow to adopt technology solutions that would voluntarily be used by consumers to facilitate contact tracing, both due to privacy concerns and uncertainty regarding whether technology is an effective replacement for the customary human-to-human contact involved in contact tracing^61^. Google and Apple—typically fierce competitors—joined together to enable apps to use Bluetooth proximity data to facilitate privacy-preserving contact tracing^63^. However, questions have arisen both about whether such information can be collected in a way that responds to privacy concerns^64^ and whether privacy controls will create unnecessary barriers to deploying these technologies in an optimal way to fight the pandemic^65^. Others have expressed concerns regarding the equitable collection and use of this information given disparities in use of smart phones and access to broadband^64^.

The collection and use by public health authorities of geolocation data for purposes of COVID-19 response illustrates the need for objective review of health-relevant data sharing. The collection of geolocation data has long been controversial^66^. Sharing such data with governments raises concerns about how those data could be used to harm individuals (such as by stigmatizing or unjustly penalizing those who are determined, based solely on this data, to be ill or at risk to themselves or others). Further, public health authorities typically are not “covered entities” under HIPAA, and laws governing how local authorities can access, use, and disclose data may vary by state and locality^67^ (Of note: the U.S. Centers for Disease Control and Prevention is covered by the federal Privacy Act of 1974 (see Supplementary Table 1)).

### Current federal data privacy bills

Several bills have been introduced in the 116^th^ Congress to fill gaps in U.S. privacy law (See Supplementary Table 2 for a sample list of those bills). For the most part, the federal bills adopt one or more of the following approaches:

- Requirements to provide individuals with clear notice about how their personal information is collected, used, and disclosed;
- Requirements to provide individuals with choices (either opt-in or opt-out) for the collection, use, and disclosure of their personal information.
- Broad definitions of personal data, with stricter standards for data to be considered to be de-identified (and therefore no longer covered).
- Establishment of individual rights concerning data, including the right to know whether a company possesses your data, the right to request corrections, the right to obtain copies, and the right to have data deleted.
- Increased authority to, and resources for, the FTC to enforce new privacy mandates; and
- Exemptions from new law for entities already covered by HIPAA.

These bills incorporate many of the customary provisions found in privacy laws but have the following key limitations, particularly for regulating the privacy of health-relevant data:

#### Too much reliance on notice and consent to protect privacy

The predominant model for protecting privacy involves companies giving individuals notice of, and rights to consent to, uses and disclosures of their data. These “commitments” regarding data are typically found in Privacy Policies and Terms of Service, and consumers are required to acknowledge that they have read and agree to these documents before they are permitted to use an app or a service.

But this model of notice and consent is widely recognized by privacy scholars as being inadequate on its own to protect privacy, particularly with respect to online transactions^68–71^. Privacy notices and terms of service are famously too long and hard to understand and are frequently missing or inadequate^72^. In an age of “big data,” it is often difficult to predict at the time of data collection all future uses^69^. Individuals too often agree to terms of service without reading them^73^. Companies design technology in ways that “maximize the collection, use, and disclosure of personal information,” challenging the notion that individuals truly can make informed choices online even when they are trying to do so^74^. Reliance on notice and consent also shifts the burden for protecting privacy to the individual, instead of holding institutions and data holders accountable for acting transparently and responsibly with individuals’ data^70^. Further, companies can change their consent policies, and consumers may not be aware of these changes or have little choice but to agree to them to continue using a service^70^. Relying on individual consent to protect privacy also fails to account for others whose interests are often implicated in health data, as some health data (such as genetic information) reveals information about family members^75^.

GDPR and new state privacy laws, such as the California Consumer Privacy Act (CCPA), tend to rely on consent (either opt-in or opt-out) for collection and use of data, particularly by commercial companies^76^. Nonetheless, these laws do not appear to have substantially limited the ubiquitous collection and use of personal data in commerce^77^.

#### Overvaluing de-identification or pseudonymization as a privacy measure

Most existing privacy laws and proposed federal bills cover only identifiable information. Consequently, information that has been de-identified, anonymized, or pseudonymized is outside of regulation. Although techniques to reduce identifiability of information lessen privacy risks, they do not reduce the risk to zero. Too often, there is no legal accountability for unauthorized re-identification. For example, HIPAA’s de-identification standard requires data to be at “very low” (not zero) risk of re-identification. Consequently, some risk of re-identification remains, but regulators cannot hold recipients of de-identified data accountable for unauthorized re-identification^78^.

More recent privacy laws, such as GDPR and CCPA, appear to have more robust standards for how data qualify as “de-identified” or pseudonymized and no longer subject to regulation. For example, under the CCPA, data that can be linked to a particular person or household, such as through an IP address or advertising identifier, is considered to be covered even if the particular individual is not identified^79^. But because the CCPA is new, it is unclear whether these definitions will be effective at giving consumers more control over robust collection and commercialization of personal data. Further, medical researchers depend on the ability to collect and analyze de-identified data. Amendments have been proposed to the CCPA to assure that the CCPA’s more stringent definition of de-identified data does not create obstacles to the collection and use of information for health and medical research purposes^80^.

#### Absence of provisions to assure availability of health data for a learning health system

Responsible collection and analysis of health-relevant data are critical to addressing deficiencies in U.S. health and health care. De-siloed data combinable for delivery, research, and public health are needed for coordinated care^81^, genomic diagnosis^82^, including accurate diagnoses across genetic ancestries^83^, comparative effectiveness research^84^, post-marketing surveillance^85^, data-driven accrual to clinical trials^86^, rare disease research (https://www.rarediseasesnetwork.org/researchers/nih-data-sharing), public health surveillance^87^, early disease detection^88^, development of digital biomarkers to manage patients care at home^89^ or to combat a pandemic^90^, and advancing discovery^91^. Sometimes inclusion of entire populations is necessary to ensure generalizability of conclusions across diverse patients and to avoid the nonrandom statistical biases that would emerge from opt-in models.

The National Academy of Medicine (then the Institute of Medicine) first proposed a Learning Healthcare System framework in 2007^1^, but progress has been slow, in part due to difficulty in accessing and sharing health-relevant data. Data to improve health and health care needs to include data sources outside of HIPAA, as much of what happens to influence an individual’s health and wellbeing occurs outside of the doctor’s office or hospital^92^. However, most of the proposed bills focus disproportionately on protecting personal data and do little to promote its availability. This shortcoming may be of little import for data not used for health purposes, but it has significant implications for health-relevant data. Ultimately, the U.S. will need a long-term, national solution that both addresses privacy and data availability. Survey data reveal that individuals practice “privacy-protective” behaviors such as not seeking health care or hiding the truth about health conditions if they don’t trust that their information will be kept confidential^93^.

### Recommended protections for health-relevant data to fuel a learning health system

Determining whether there are sufficient protections for data based on whether an entity is or is not covered by HIPAA arguably is no longer the appropriate benchmark. The lack of strong, consistent protections for health data that respond to 21st-century risks could have the “long term effect of reducing the uptake of new innovative technologies” and undermining the promise of digital medicine^18^. At the same time, focusing just on privacy without assuring needed data flows fails to address the compelling need for data to address significant health needs^94^, including the need to address significant disparities in health outcomes based on race and gender. Creation of a learning health system may require a “moral priority on learning,” with active contributions of data from both health care professionals and from patients^95^.

The dual needs in health to both protect individuals and assure data availability to improve individual and population health call for comprehensive policies governing all entities collecting and using health-relevant information whether covered by HIPAA or not. Policymakers need not reinvent the wheel and can draw from HIPAA’s framework, as well as FTC recommendations. Specifically, in 2012, the FTC issued a report, Protecting Consumer Privacy in an Era of Rapid Change (hereinafter “FTC Report”)^96^. The report established recommendations for privacy “best practices” to be adopted by all commercial companies, except smaller companies and those not sharing sensitive data with third parties^96^. Though the report was not focused on health-relevant data and is now eight years old, the best practice recommendations nonetheless provide some noteworthy approaches for establishing enforceable rules and norms for this data.

U.S. policymakers should consider these recommendations in crafting privacy bills. But given the glacial pace of federal legislation, the recommendations below also can inform best practices adopted by companies in the absence of any new federal requirements. These best practices, if publicly attested to by companies already covered by the FTCA, can be enforced by the FTC (such as if a commercial company publicly commits to a data limitation (for example, not sharing data except with consent) but doesn’t actually follow that practice)^96^.

#### Establish rules for health-relevant data rather than relying just on consent

Although HIPAA has its deficiencies, its overall comprehensive approach has value in considering how to govern health-relevant data, even when collected and used outside of the health care system. For example, HIPAA’s regulations include a role for individual consent but do not push all of the obligations for protecting privacy to the individual, instead creating enforceable boundaries for when and how identifiable information can be used and shared. From its inception, HIPAA’s regulatory framework has recognized that health data must be protected and also made available for treatment, to secure payment, to enable health care institutions and medical practices to conduct operations, for public health, and research purposes.

On the other hand, HIPAA’s drafters established a comprehensive list of required and permitted uses and disclosures to enable data flows typical in a functioning health care system (see Supplementary Discussion); that list would not necessarily effectively govern data collected, used, and shared by commercial companies outside of healthcare. Lawmakers will need to establish a list of permitted collections, uses, and disclosures that more directly address the privacy risks in the commercial space. Also, there are few, if any, prohibitions on what an entity covered by HIPAA can do with data, as uses or disclosures not expressly permitted can still occur with the written authorization of the individual. To effectively govern commercial companies’ behavior with health-relevant data, lawmakers will need to prohibit uses and disclosures where the privacy risks are significant in comparison to the benefits.

#### Impose collection, use, and disclosure limitations

In the FTC Report, the Commission recommended that “companies should limit data collection to that which is consistent with the context of a particular transaction or the consumer’s relationship with the business, or as required or specifically authorized by law.”^96^ In other words, data collection should be limited to what a consumer might expect, given the context. “Fair Information Practice Principles,” the foundation for information privacy law, include collection limitations as a critical component of protecting data^97^.

However, the concept of “collection limitations” may seem antithetical to the robust health data enterprise that contributes to a learning health system. HIPAA’s regulations contain few limits on whether entities may collect health information, choosing instead to comprehensively regulate how that information can be used and disclosed once an entity covered by HIPAA has it. When HHS first drafted the HIPAA regulations, it may have made sense to disregard collection limitations. HHS was setting ground rules for how a defined set of entities within the health care system could handle data. But as health care entities increasingly collect data on socioeconomic determinants, in some cases beyond what patients might expect^98^, policymakers may want to consider whether collection limitations should be imposed in HIPAA (for example, requiring such collection to be directly connected to addressing individual or population health).

Further, for commercial companies, whose business models revolve around monetization of personal information, some limits on the collection of health-relevant data make sense. For example, the collection of health-relevant data could be prohibited unless the data collection is consistent with consumer expectations and intended to benefit the individual or population health. For example, a bill drafted (but not yet introduced) by Senator Sherrod Brown (D-OH) would prohibit the collection of personal data unless it is “strictly necessary” to provide the good or service sought by the consumer^77^.

Use and disclosures of health-relevant data similarly should be limited to what the consumer would reasonably expect, given the context^70^. This maxim also should govern the repurposing of information. For example, technology and telecommunications companies routinely collect geolocation data (https://www.gravitatedesign.com/blog/what-is-geolocation/). Today, governments across the world are seeking or already collecting these data for COVID-19 response activities. These data were not collected for this purpose initially, and consumers likely did not expect it to be used for this purpose. The FTC Report recommends consumer consent be obtained before collecting, using, or disclosing information in ways not consistent with the context of consumer’s relationship to the company^96^. Given the limitations of relying on consent for protecting privacy, such an approach feels ripe for abuse. Individual consent should be required, but some additional gating criteria may be needed to rein in companies’ tendencies to pursue uses and disclosures of data that enhance the bottom line, do not contribute to improving individual and/or population health, and where the impact of risks to privacy created by those activities is chiefly borne by the consumer.

#### Assure beneficial uses of health-relevant data

Federal policies should also assure that data are available to be used ethically to address health system improvements. Of note, one of the federal bills pending before Congress - the Data Care Act^99^ would impose duties of “care, loyalty, and confidentiality” on online service providers that collect individual identifying information, detailing specific requirements and prohibitions under each of the three categories (See Supplementary Discussion). This approach is appealing for governing health-relevant data—but the categories are so broadly worded that it is unclear it would result in beneficial uses of these data consistently across all data holders. Though allowable under HIPAA, the sale of “de-identified” data by covered entities is another flashpoint in an expanding debate^100^ which suggests that policies governing health-relevant data should address de-identified as well as identifiable data.

Companies collecting, using or disclosing health-relevant data (identifiable and de-identified) could be required to establish independent data ethics review boards. Such boards would evaluate proposed data projects for legal and ethical implications as well as the potential to improve health or the health care system [101]. Such boards could be similar to Institutional Review Boards (IRBs), which provide ethical review of proposals for research on human subjects under the federal Common Rule^102^. However, data ethics review boards should focus more on privacy than interventional risk and include members with substantial privacy expertise. Today, the Common Rule does not require IRB review of research using data that are not identifiable and provides exemptions (including rapid review by one or two members of the IRB) for research using identifiable data^103^. Further, data ethics review boards could evaluate uses and disclosures beyond just those for research.

This approach provides no magic bullet. Notably, IRBs have not been a panacea for assuring the ethical conduct of human subjects research. The proposed data ethics review boards similarly would need to be established with safeguards against industry capture and conflicts of interest and should not be viewed as a comprehensive solution. For such boards to be effective, they must have independence from the company and ideally include outsiders, such as consumers and experts. Facebook recently announced the establishment of an independent Oversight Board to achieve “fair decision-making” concerning the removal of unacceptable content on the site. Among the Board’s authorities are to “instruct” Facebook to allow or remove content and “interpret” Facebook’s Community Standards and other policies “in light of Facebook’s articulated values”^104^. Of note, Facebook relied on a provision in Delaware law, the Purpose Trust Statute, to assure the Board was independent of Facebook^105^. However, notwithstanding this unique (and expensive) endeavor by Facebook, there is little evidence that this Board has made a difference in assuring better decision-making with respect to content on the site^106^. Similarly, Google dismantled its ethics board intended to “guide responsible development of AI [artificial intelligence]” at Google shortly after it was established due to controversy over its membership^107^.

In another example, GDPR requires a Data Protection Impact Assessment, and in some cases regulatory review, for certain high-risk processing activities, such as health data processed in large numbers^43^. Similarly, U.S. federal agencies are required to conduct Privacy Impact Assessments “for all new or substantially changed technology” that collects, maintains, or disseminates personally-identifying information (https://www.archives.gov/privacy/privacy-impact-assessments). Such assessments could be valuable if subject to independent, objective review and not merely check-the-box exercises.

“Data trusts” or “civic trusts” also have been proposed as legal mechanisms for assuring that companies use and disclose consumers’ personal data for the benefit of consumers, even after a change in company strategy or sale of the company^108^. Consumer data trusts have been defined as “intermediaries that aggregate consumers’ interests and represent them vis-à-vis data-using organizations”^109^. By aggregating consumer interests, consumer trusts would have bargaining power to negotiate better terms of data use and disclosure than could be achieved by any individual consumer^109^. Existing laws giving individuals the right to copies of their information (for example, HIPAA, CCPA, and GDPR) could help facilitate the establishment of these trusts, as individuals could direct copies of their personal data to be held and managed therein. Common to the different versions of data trusts is the use of trust law to help assure that commitments regarding how data can be accessed, used, and disclosed are honored. But trusts also can be established to protect private interests; consequently, the ability of a data trust to assure only responsible uses of data depends on what terms and conditions are established for use and disclosure of the data, and who establishes those rules.

Another option is to require companies collecting or processing health or health-relevant data to adhere to additional oversight and requirements. Ontario province in Canada permits data custodians to disclose personal health information for health system improvement purposes. However, custodians may disclose information only to entities approved by the Privacy Commissioner as having adequate practices and procedures to protect privacy and maintain confidentiality^110^. In a variation on that theme, health data collection and processing could be limited only to entities that demonstrate (through periodic audits) that they meet ethical, privacy, and security standards. Companies collecting health-relevant data also could be required to segment or “firewall” their health business from other aspects of the company.

Health system entities customarily rely on “data use agreements” to bind recipients of health data to contractual commitments regarding the use of that data. Such agreements often include prohibitions on further uses and disclosures and, in the case of de-identified information, commitments not to re-identify individuals in the dataset. HIPAA requires a data use agreement when a “limited data set” (data stripped of 16 common identifiers) is used or disclosed for routine health care operations, public health, or research^111^. Data use agreements also may be voluntarily adopted when sharing even de-identified data, as an additional measure of protection adopted by the disclosing entity. However, such agreements are not a scalable solution to protecting health-relevant data. They depend on the parties to the contract to agree to responsible terms (often difficult where the data recipient has greater bargaining power), and those terms can only be enforced by the parties to the contract. While such contracts can be protective, they can also be vehicles for protecting data as a proprietary asset, which can limit the availability of data even for potentially beneficial uses.

#### Increase transparency and choice around health data uses and disclosures

Although notice and consent should not be the cornerstone for privacy, individuals still want and should have notice of, and some choice about, collection, use, and disclosure of health-relevant information (https://ogury.com/blog/how-consumers-really-feel-about-their-privacy-and-data/). The FTC Report called for “simplified choice,” with clearer, shorter, and more standardized privacy notices, in circumstances where the data collection, use, and sharing is beyond what consumers would ordinarily expect or involves sensitive data^96^. For example, companies can improve notice and choice through layered notice and use of visuals to improve comprehension^112,113^.

But even if consent is not sought for a particular use or disclosure, either because it is within consumer expectations or is mandated or authorized by law, companies should still be required to be transparent about data uses and disclosures (https://bankingjournal.aba.com/2019/06/study-consumers-increasingly-concerned-with-data-security-privacy/). As demonstrated by the public reaction to Google’s arrangements with Ascension Health System, greater transparency of health data uses and disclosures appears important to engendering public trust in digital medicine technologies.

Even within traditional health care, there is a need for greater communication about health-relevant data uses and disclosures. The National Research Council report on Precision Medicine emphasizes that it is patients who “uniquely understand the potential value of a social contract in which patients both contribute personal clinical data and benefit from the knowledge gained through the collaboration”^114^. In consenting to care and treatment, physicians, hospitals, and health systems should consider entering a compact with patients such that data and biospecimens captured as a byproduct of the care delivery system can be aggregated and linked to be used in a learning health system. Both consent to treat documents and the notice of privacy practices provided to patients should explicitly outline the compact^82^.

#### Strengthen remedies for harms

Ideally, protections for health-relevant data should go beyond addressing privacy and also address the potential for harm. Historically, in the U.S. policymakers have separated addressing discrimination—such as through the enactment of provisions in the Affordable Care Act prohibiting discrimination in health insurance based on health status or history—and privacy. However, in many respects privacy and nondiscrimination can collectively help create public trust in the collection, use, and sharing of health-relevant data^115^. The Genetic Information Nondiscrimination Act (GINA) is one model of a combined privacy and antidiscrimination law^116^. For example, GINA prohibits employers from collecting genetic information (protecting privacy) and prohibits employers from discriminating based on genetic information. Because health-relevant data can be collected and used for benefit and harm (compare using information to target scarce health care resources toward individuals and populations most in need with using that same information to avoid enrolling individuals and populations likely to be more costly and difficult to treat), it is critically important that policies not just focus on controls on health-relevant data and also address and minimize the opportunities for information to be used in ways that harm individuals and populations.

What may be needed are stronger protections against discrimination—for example, discrimination in health and health-related insurance (for example, disability insurance) and protections against harmful employer uses of health-relevant information. The Affordable Care Act, which prohibits discrimination in the provision of health insurance (except with respect to information on smoking status) is perceived to be on shaky ground due to persistent opposition from a number of Republican policymakers (https://www.bbc.com/news/world-us-canada-24370967), and there are no federal protections for disability and life insurance. Similarly, the Americans with Disabilities Act provides employment protections for individuals with disabilities—but these protections do not extend to health information collected about persons who do not meet the definition of an individual with a disability^117^.

In protecting against potential “harms” of data use, policymakers also need to consider the chilling effect that law enforcement access to data will have on the willingness of individuals—particularly marginalized populations—to have their data collected and used for “learning” purposes. Data for Black Lives is “committed to the mission of using data science to create concrete and measurable change in the lives of Black people,” recognizing that data has “tremendous potential to empower communities of color”—while at the same time data is too often “wielded as an instrument of oppression, reinforcing inequality and perpetuating injustice.” (https://d4bl.org/about.html) Privacy laws often contain exemptions for access to data by law enforcement. For example, HIPAA allows law enforcement access to data based on an “administrative request” if the information sought is “relevant and material to a legitimate law enforcement inquiry,” limited in scope, and requiring identifiable (vs. de-identified) information^118^. HIPAA also allows entities to release certain medical information to law enforcement—including name, address, blood type, and physical characteristics—to identify or locate a suspect, fugitive, witness or missing person^119^. For entities not covered by HIPAA, company commitments may provide the only assurance that law enforcement access to data will be required to meet probable cause standards, as determined by a neutral authority (such as a court), absent stronger protections in applicable state law. Police recently caught the Golden State Killer by matching DNA found at a crime scene with DNA in a free online genetic database used by one of his relatives. Although this information helped police to resolve 12 murders and at least 45 rapes committed in California between 1976 to 1986, the potential for law enforcement access to online health-relevant databases may deter individuals from using these tools^120^.

In developing its 2012 report, the FTC expressly rejected calls for a “harm-based” model of privacy that focuses only on protecting consumers from harms like “physical security, economic injury, and unwanted intrusions into their daily lives.”^96^ FTC’s contention was that such a model would fail to recognize “a wider range of privacy-related concerns, including reputational harm or the fear of being monitored.”^96^ Feelings of risk and anxiety also are among the harms suffered by individuals whose data are breached^121^. Rules-based privacy regimes like HIPAA instead create enforceable expectations regarding how health data must be handled without regard to whether or not individuals or populations suffer any cognizable harm when organizations don’t follow the rules. In addition to addressing discrimination harms, policymakers should also consider addressing more traditional privacy harms (for example, breaches of heath information). In enforcing HIPAA, OCR considers whether a HIPAA violation harmed individuals in determining the level of civil monetary penalty it will pursue^122^. Through HITECH, Congress amended the HIPAA Privacy Rule to require HHS to establish a mechanism to enable individuals “harmed” by HIPAA violations to receive a portion of any civil monetary penalties or settlements imposed or reached by HHS. However, HHS has yet to act on this measure^13^.

Harm should not be the linchpin of privacy regulation; but addressing harm should be a component, particularly for health-relevant data given its sensitivity. One interesting example is a privacy tax on data collectors and processors that could fund a no-fault compensation program for privacy harms^123^. Companies also could be required to establish funds to compensate harms, with broad recognition of the types of privacy harms that can occur due to unauthorized or unethical uses or disclosures of data^123^.

#### Maintain incentives to use and disclose data which are less identifiable—but refrain from treating these data as zero risk

In general, information at low or very low risk of re-identification is typically not subject to privacy laws and consequently is not regulated. Such an approach leaves privacy risk on the table; however, relaxing regulations on data at very low risk of re-identification provides incentives for entities to collect, use, and disclose data with fewer privacy risks. Experience with HIPAA’s rules for de-identification suggests that if the law sets clear and achievable standards for de-identification, entities will leverage de-identified data for public health, research, and business analytics. On the other hand, anger and frustration over commercialization of HIPAA de-identified health data appears to be increasing—and some entities are responding to those concerns^124^. For example, one renowned medical center has recently adopted an ethical framework for sharing even de-identified data and biospecimens with external entities, including commercial companies^125^.

Regulation of health-relevant data should provide incentives for the use and disclosure of that data in less identifiable forms. However, given that this data will still retain some residual risk of re-identification, this data should be subject to some regulation. For example, civil monetary penalties should be imposed for unauthorized re-identification of de-identified data and criminal penalties for intentional re-identification. But merely controlling for risk of re-identification will not be sufficient to garner consumer trust in how companies handle their health-relevant data, even if it is “de-identified.” Companies should be required to be transparent about the uses and disclosures of de-identified data and to identify the general methodologies used for de-identification. Because consent is not sufficiently protective of privacy, uses and disclosures of de-identified data also could be subject to ethics board review.

## Conclusion

To fully realize the potential of digital data and digital medicine, and to advance U.S health and healthcare toward becoming a rapid learning system, the U.S. will need comprehensive privacy and security protections for health data regardless of where the data are collected or maintained. At the same time, such health protections must also encourage responsible uses and disclosures. The COVID-19 pandemic shines a spotlight on this problem—but COVID-19 is far from the only health issue that more robust access to data could help address. And in the aftermath of COVID-19, as health threats ease, re-equilibrating around access to health care data will be an essential conversation. To date privacy bills introduced to date focus more on protecting health-relevant data than on assuring its appropriate use. Also, proposed measures for protecting data rely too much on notice and consent and de-identification of data as protections.

What is needed is a multi-pronged approach that implements strong privacy protections but also includes accountability even for uses of so-called “de-identified” or anonymized data and addresses the potential for harm to individuals and populations. Such measures should also facilitate assure the availability of health-relevant data for societal benefit and to support a learning healthcare system Such a transformation could not be more important and urgent, as U.S. healthcare adapts to the impact of a global pandemic, struggles to care for an aging population, faces a diminishing primary care workforce, and continues to have the highest expenditures in the world despite often having poorer health outcomes. Innovative and pervasive use of data must underpin any substantial transformation.

## Supplementary information

Supplementary Information

## Data Availability

This paper is not original research involving data collection. Hence, there is no research data to make available.
