# Supplementary material for: Privacy protections to encourage use of health-relevant digital data in a learning health system
Source: NPJ Digit Med. 2021 Jan 4;4:2. doi: 10.1038/s41746-020-00362-8 (PMC7782585; doi:10.1038/s41746-020-00362-8)
Supplement: Supplementary file 1 — Supplementary Information [file 41746_2020_362_MOESM1_ESM.pdf]

## Supplementary Discussion

Overview of Key Laws Protecting the Privacy of Health Data within the Health Care System

**HIPAA.** HIPAA's privacy, security, and breach notification regulations are the most commonly applied and most comprehensive set of health-relevant data privacy protections in the U.S. HIPAA governs a wide range of identifiable "protected health information" (PHI), which is broadly defined and includes demographic and other information related to current or past health status that is created, held, or transmitted by an entity covered by HIPAA.[1] However, in terms of "who" is covered by the law, its scope is narrow. In essence, it applies to “covered entities” which transmit information governed by Department of Health and Human Services standards. This includes most health care providers, health plans and health care clearinghouses. HIPAA also applies to their contractors (known as “business associates”).[2] Of note, because the HIPAA statute was enacted to facilitate standardized electronic billing between health care providers and health plans, it does not cover health care providers who do not accept any health insurance.[3] An example of such a provider is a concierge medical practice which is paid for solely by the consumer out of pocket.

When HIPAA applies, its Privacy Rule includes detailed provisions regarding how PHI, in digital, paper, or other forms, can be used and disclosed. HIPAA expressly permits beneficial uses and disclosures, such as for treatment, payment, healthcare operations, public health, and research.[4] For research, individual authorization is required unless waived by a Privacy Board or Institutional Review Board (IRB).[5] Any uses or disclosures not expressly permitted by the Privacy Rule require the prior authorization of the data subject.[6] HIPAA also establishes rights for individuals, including the right to obtain a copy of PHI and to request amendments to this data.[7] The Security Rule establishes baseline physical, technical, and administrative safeguards that apply to electronic PHI,[8] and the Breach Notification Rule requires notification of individuals and regulators in the event of breaches of PHI.[9] HIPAA also defines de-identified data and sets standards on how to achieve it, but places no limits on its use or disclosure, regardless of who controls the information.[10]

**HITECH.** In 2009, Congress enacted the Health Information Technology for Economic and Clinical Health Act (HITECH), which made a number of changes to the HIPAA Privacy Rule.[11] Important changes include:

- Establishing breach notification requirements for entities covered by HIPAA,
- Making business associates directly accountable to regulators for compliance with the HIPAA Security Rule and select components of the Privacy Rule,
- Prohibiting the sale of identifiable information without individual authorization,
- Increasing penalties for violations of HIPAA regulations, and
- Clarifying the definition of marketing and requiring entities to obtain authorization for using or disclosing an individual’s identifiable information for marketing purposes.[12]

**Substance Abuse Treatment Regulations**. Commonly referred to as “Part 2” due to their location in the Code of Federal Regulations,[13] these regulations protect identifiable information

collected, used, and disclosed by federally supported substance abuse treatment programs.[14] In general, the regulations require authorization from the individual before data covered by these regulations can be shared with a third party.[15] Recipients of these data must also not reuse or redisclose this information without individual authorization.[16]

**The Common Rule**. This rule governs federally supported human subjects research, which includes research using identifiable personal information.[17] In general, informed consent of the individual is required before their information can be used in research; however, an IRB can waive this requirement (using similar criteria to those required for a HIPAA waiver).[18] New revisions to the Common Rule also allow entities covered by HIPAA to rely solely on HIPAA to govern research using identifiable information.[19] In addition, broad consent is permitted to create multi-use research databases. Notably, IRB approval of research using identifiable data is required even if consent is not required to be sought.[20]

<u>Permitted Uses and/or Disclosures under the HIPAA Privacy Rule w/out the need to obtain consent or authorization</u> (and in most cases, notwithstanding the objection of the individual)

- Treatment (45 CFR §164.502(a)(1)(ii) & §164.506(a))
- Payment and payment-related activities 45 CFR §164.502(a)(1)(ii) & §164.506(a)
- Health care operations: (45 CFR §164.500, §164.502(a)(1)(ii), and §164.506(a)
    - Quality assessment and improvement activities
    - Population-based activities relating to improving health or reducing costs
    - Case management and care coordination
    - Reviewing the competence of health care professionals, evaluating provider performance, training of health care professionals, and licensure/certification/accreditation activities
    - Underwriting and other activities related to health insurance
    - Medical review, legal, and auditing, including fraud and abuse detection and compliance
    - Business planning and development
    - Business management and general administrative activities (including fundraising for the benefit of the covered entity and sale or transfer of covered entity assets)
- To public health authorities for public health purposes (45 CFR §164.512(b))
- To business associates (and sub-business associates (provided a HIPAA-compliant business associate agreement (BAA) is executed) (45 CFR §164.502(e)(1))
- Where required by other law (such as a state law mandating disclosure of health information) (45 CFR §154.512(a))
- Health care oversight (to health oversight agencies) (45 CFR §164.512(d))
- To avert a serious threat to health and safety (45 CFR §164.512(j))
- As part of judicial and administrative proceedings (45 CFR §164.512(e))
- For disaster relief (to disaster relief organizations) (45 CFR §164.510(b)(4))
- Law enforcement (subject to conditions) (45 CFR §164.512(f))

- For national security (45 CFR §164.512(k)(2))
- Disclosures about victims of abuse and neglect (45 CFR §164.512(c))
- For tissue or organ donation purposes (45 CFR §164.512(h))
- To coroners, medical examiners, funeral directors (45 CFR §164.512(g))
- For research, if the need for the data subject's authorization is waived by an Institutional Review Board or Privacy Board (45 CFR §164.512(i))

**Supplementary Table 1: Other Federal Data Protection Laws**[21]

| Federal Law | Entities Covered by Law | Type of Information Covered | General Requirements |
|---|---|---|---|
| Children’s Online Privacy Protection Act (15 U.S. Code 6501-6506) | Websites or online services directed to children (or where there is knowledge they are collecting identifiable information from a child) | Identifiable information collected online from a child under age 13 | Prohibits online collection of information from children without parental consent; requires publication of privacy notices. |
| The Communications Act) (47 U.S. Code 222(c)) (including amendments by the Telecommunications Act of 1996) | Common carriers; cable operators and satellite carriers | Customer Network Proprietary Information (CPNI) & personally identifiable information | Prohibits use or disclosure of individually identifiable CPNI without customer approval (with exceptions); requires implementation of safeguards to ensure proper use and disclosure of CPNI; requires notification in the event of breach. |
| Computer Fraud and Abuse Act (18 U.S. Code 1030) | All | Information in a “protected computer” (any computer used in or affecting interstate commerce or communications – i.e., connected to the Internet) | Prohibits intentionally accessing and obtaining information from a computer without authorization. |
| Electronic Communications Privacy Act (three separate components: Wiretap Act, Stored Communications Act, and Pen Register Act) | Much of ECPA covers law enforcement but other provisions apply to nongovernmental actors | -Wire, oral, or electronic communications in transit<br>-Stored electronic communications<br>-Meta data regarding communications | Prohibits: intentional interception of wire, oral, or electronic communications; improper access or disclosure of certain electronic communications in storage; installation of a “pen register” (tracing device) without a court order. |

| Federal Law | Entities Covered by Law | Type of Information Covered | General Requirements |
|---|---|---|---|
| (18 U.S. Code 2510-3127) | | | |
| Fair Credit Reporting Act (15 U.S. code 1681) | Consumer reporting agencies, users of reports generated by such agencies | Consumer reports | Restricts use of information regarding an individual's credit worthiness; requires truncating credit card numbers on receipts; regulates certain marketing uses of information; also mandates programs to detect and respond to identity theft. |
| Family Educational Rights and Privacy Act (FERPA) (20 U.S. Code 1232g) | Educational agencies or institutions receiving federal funds | Identifiable education records | Gives students the right to inspect and revise student records for accuracy; prohibits disclosure of records or other personal information on a student without the student or parent's consent. |
| Gramm Leach Bliley Act (15 U.S. Code 6801-6809) | Banks, insurance companies, other companies in financial services | Nonpublic personal information (NPI) | Imposes requirements for securing NPI; restricts use and disclosure of NPI; requires notification in the event of breach. |
| Telephone Consumer Protection Act (TCPA) (47 U.S. Code 227) | Entities sending automated text messages or making automated calls | Automated calls and text messages made for marketing purposes | Requires opt-in consent prior to sending initial message or making initial call; requires clear opt-out for text messages. |
| Video Privacy Protection Act (18 U.S. Code 2710 et. seq.) | Videotape service providers | Personally identifiable information re: videotape sales and rentals | Protects wrongful disclosure of videotape rental or sale records, or similar audio-visual materials, including online streaming. |
| The Privacy Act of 1974 (5 U.S. Code 552a) | Federal government agencies | Personally identifiable information in a federal system of records | Requires agencies to provide public notice of their systems of records; requires individual consent for disclosure of personally identifiable information, subject to 12 exceptions.[22] |

**Supplementary Table 2: Federal Data Privacy Bills – 116th Congress**

As of May 9, 2020, a search for "data privacy" in GovTrack.us (www.govtrack.us), a website that tracks legislation in the U.S. Congress, yielded 2,566 bills introduced in the 116th Congress (2019-2021). The first 15 on the list are described briefly below. (Bills beginning with S are those introduced in the Senate; bills beginning with H.R. are those introduced in the House of Representatives.) All information below is current as of the date this paper was submitted for publication.

| Bill Number and Name | Sponsor(s) | Bill Description | Status |
|---|---|---|---|
| S.583, DATA Privacy Act | Senator Cortez Masto (D-NV) | "To provide for digital accountability and transparency." | Referred to Committee on Commerce, Science, and Transportation |
| S.3456, Consumer Data Privacy and Security Act of 2020 | Senator Moran (R-KS) | "To protect the privacy of consumers." | Referred to Committee on Commerce, Science, and Transportation |
| S.3663, COVID-19 Consumer Data Protection Act of 2020 | Senators Wicker (R-MS), Thune (R-SD), Moran (R-KS), Blackburn (R-TN), Fischer (R-NE). | "To protect the privacy of consumer's personal health information, proximity data, device data, and geolocation data, during the coronavirus public health crisis." | Referred to the Committee on Commerce, Science, and Transportation. |
| S.3300, Data Protection Act of 2020 | Senator Gillibrand (D-NY) | "To establish a Federal data protection agency, and for other purposes." | Referred to Committee on Commerce, Science, and Transportation |
| S.2968, Consumer Online Privacy Rights Act | Senators Cantwell (D-WA), Schatz (D-HI), Klobuchar (D-MN), Markey (D-MA). | "To provide consumers with foundational data privacy rights, create strong oversight mechanisms, and establish meaningful enforcement." | Referred to Committee on Commerce, Science, and Transportation |
| S.1842, Protecting Personal Health Data Act | Senators Klobuchar (D-MN) and Murkowski (R-AK) | "To protect the personal health data of all Americans." | Referred to Committee on Health, Education, Labor, and Pensions |

| Bill Number and Name | Sponsor(s) | Bill Description | Status |
|---|---|---|---|
| S.189, Social Media Privacy Protection and Consumer Rights Act of 2019 | Senators Klobuchar (D-MN) and Kennedy (R-LA) | "To protect the privacy of users of social media and other online platforms." | Referred to Committee on Commerce, Science, and Transportation |
| H.R.2013, Information Transparency & Personal Data Control Act | Representatives DelBene (D-WA), Rice (D-NY), and Suozzi (D-NY) | "To require the Federal Trade Commission to promulgate regulations related to sensitive personal information, and for other purposes." | Referred to the Committee on Energy and Commerce |
| H.R. 4978, Online Privacy Act of 2019 | Representatives Eshoo (D-CA) and Lofgren (D-CA) | "To provide for individual rights relating to privacy of personal information, to establish privacy and security requirements for covered entities relating to personal information, and to establish an agency to be known as the United States Digital Privacy Agency to enforce such rights and requirements, and for other purposes." | Referred to the Committee on Energy and Commerce and to the Committee on the Judiciary |
| S.2398, Voter Privacy Act of 2019 | Senator Feinstein (D-CA) | "To amend the Federal Election Campaign Act of 1971 to ensure privacy with respect to voter information." | Referred to the Committee on Rules and Administration |
| S.847, Commercial Facial Recognition Privacy Act of 2019 | Senators Blount (R-MO) and Schatz (D-HI) | "To prohibit certain entities from using facial recognition technology to identify or track an end user without obtaining the affirmative consent of the | Referred to Committee on Commerce, Science, and Transportation |

| Bill Number and Name | Sponsor(s) | Bill Description | Status |
|---|---|---|---|
| | | end user, and for other purposes.” | |
| S.1214, Privacy Bill of Rights Act | Senator Markey (D-MA) | “To establish and protect individual and collective privacy rights, and for other purposes.” | Referred to Committee on Commerce, Science, and Transportation |
| S.2961, Data Care Act of 2019 | Senators Schatz (D-HI), Bennet (D-CO), Cortez Masto (D-NV), Markey (D-MA), Duckworth (D-IL), Baldwin (D-WI), Manchin (D-WV), Durbin (D-IL), Brown (D-OH), Booker (D-NJ), Klobuchar (D-MN), Hassan (D-NH), Heinrich (D-NM), Murray (D-WA), Sanders (I-VT), Murphy (D-CT) | “To establish duties for online service providers with respect to end user data that such providers collect and use.” | Referred to Committee on Commerce, Science, and Transportation |
| S.2889, National Security and Personal Data Protection Act of 2019 | Senators Hawley (R-MO), Cotton (R-AR), Rubio (R-FL) | “To safeguard data of Americans from foreign governments that pose risks to national security by imposing data security requirements and strengthening review of foreign investments, and for other purposes.” | Referred to Committee on Commerce, Science, and Transportation |
| S.2885, Stop Marketing And Revealing The Wearables And Trackers Consumers Health Data Act | Senators Cassidy (R-LA) and Rosen (D-NV) | “A bill to prohibit the transfer or sale of consumer health information, and for other purposes.” | Referred to Committee on Health, Education, Labor, and Pensions |

1

1 References

[1] Code of Federal Regulations title 45, § 160.103.
[2] U.S. Department of Health and Human Services. Summary of the HIPAA Privacy Rule. https://www.hhs.gov/sites/default/files/privacysummary.pdf (May 2003).
[3] Id.
[4] Id.
[5] Id.
[6] Id.
[7] Id.
[8] U.S. Department of Health and Human Services. Summary of the HIPAA Security Rule. https://www.hhs.gov/hipaa/for-professionals/security/laws-regulations/index.html (accessed June 18, 2020).
[9] U.S. Department of Health and Human Services. Breach Notification Rule. https://www.hhs.gov/hipaa/for-professionals/breach-notification/index.html (July 26, 2013).
[10] U.S. Department of Health and Human Services. Summary of the HIPAA Privacy Rule. https://www.hhs.gov/sites/default/files/privacysummary.pdf (May 2003).
[11] Health Information Technology for Economic and Clinical Health Act (HITECH). Public Law No. 111-5, 123 Stat. 226 (Feb. 17, 2009).
[12] U.S. Department of Health and Human Services. HITECH Act Rulemaking and Implementation Update. https://www.hhs.gov/hipaa/for-professionals/security/guidance/hitech-act-rulemakingimplementation-update/index.html (July 26, 2013).
[13] Code of Federal Regulations title 42 part 2.
[14] HHS Office of the National Coordinator for Health IT and Substance Abuse and Mental Health Services Administration. Disclosure of Substance Abuse Disorder Patient Records: How Do I Exchange Part 2 Data? https://www.samhsa.gov/sites/default/files/how-do-i-exchange-part2.pdf (accessed June 18, 2020).
[15] Id.
[16] Id.
[17] Code of Federal Regulations title 45 part 46.
[18] Menikoff, J., Kaneshiro, J., Pritchard, I. The Common Rule, Updated. *New Engl J Med.* **375(7)**, 613-615 ((Jan. 19, 2017).
[19] Id.
[20] Id.
[21] Congressional Research Service. Data Protection Law: An Overview. https://fas.org/sgp/crs/misc/R45631.pdf
(March 25, 2019); Chabinsky, S. & Pittman, F.P. USA: Data Protection 2019. https://iclg.com/practice-areas/data-protection-laws-and-regulations/usa#:~:text=There%20is%20no%20single%20principal,personal%20data%20of%20U.S.%20residents. (March 7, 2019).
[22] U.S. Department of Justice. Privacy Act of 1974. https://www.justice.gov/opcl/privacy-act-1974#:~:text=Privacy%20Act%20of%201974,of%20records%20by%20federal%20agencies.
(accessed on June 23, 2020).
